# Impact of Prospective Audit and Feedback Antimicrobial Stewardship Strategy: A Retrospective Analysis in a Tertiary Hospital in Colombia

**DOI:** 10.7759/cureus.106129

**Published:** 2026-03-30

**Authors:** Santiago Sanchez, Edna Mora-Robayo, Sonia Reyes, Marcela Vargas, Maria Paula Morales

**Affiliations:** 1 Infectious Diseases, Hospital Universitario San Rafael de Tunja, Tunja, COL; 2 General Medicine, Unisanitas, Bogotá, COL; 3 Epidemiology and Public Health, Hospital Universitario San Rafael de Tunja, Tunja, COL; 4 General Medicine, Hospital Universitario San Rafael de Tunja, Tunja, COL

**Keywords:** antimicrobial stewardship, bacterial, beta lactam antibiotics, drug resistance, urinary tract infections

## Abstract

Background

Antimicrobial stewardship programs are fundamental strategies for improving prescribing practices. In Colombia, evidence of their impact is limited.

Objective

The objective of this study was to ascertain the role of antimicrobial therapy change, as an intervention of the antimicrobial stewardship programme, as an audit strategy, in the period from January to July 2024 in a tertiary care hospital in Colombia, Hospital Universitario San Rafael de Tunja.

Methods

Patients, including adults over 18 years, were evaluated through an audit-and-feedback stewardship strategy, while data analysis was retrospectively collected. Patients who died, were discharged, or were referred before the antimicrobial stewardship programme team intervention were excluded. Clinical, microbiological, and therapeutic characteristics, duration of treatment, and adherence to the antimicrobial stewardship programme’s recommendations were analysed.

Results

A total of 487 patients were included in the study, with a median age of 59 years and a male-to-female ratio of 53.8% to 46.2%. The primary diagnosis was urinary tract infection, accounting for 27.7% of cases. Piperacillin/tazobactam was the most commonly used empirical antimicrobial. Following the implementation of the antimicrobial stewardship programme at the hospital in January 2024, treatment was adjusted in 78.2% of cases, with de-escalation (37.8%) and stepwise escalation (37.6%) predominating. Following the interventions by the antimicrobial stewardship group, 92.2% of prescribers demonstrated overall compliance. Targeted treatment was significantly shorter in patients who underwent intervention (median: 5 days) compared to those who did not (7 days; p < 0.001). The 30-day readmission rate was 19.1%, with no significant differences between groups.

Conclusions

The implementation of antimicrobial stewardship programmes enabled the optimisation of antimicrobial therapy through individualised adjustments, achieving high adherence and a reduction in treatment duration, supporting the role of antimicrobial stewardship programmes as a key strategy for improving prescription quality. However, prospective studies are needed to evaluate its long-term clinical, microbiological, and economic impact.

## Introduction

Antimicrobial resistance (AMR) is a global problem, with an estimated 1.7 million deaths annually associated with AMR in low- and middle-income countries such as Colombia [1]. It could be related to various factors, such as resistance mechanisms, including genes or random mutations, such as the speed of replication by microorganisms, the presence of environmental reservoirs, the use of over-the-counter antibiotics, etc. However, this is not only a problem in developing countries. For example, the United Kingdom has implemented a national plan with specific strategies to combat AMR, including optimising the use of antimicrobials with the aim of reducing and controlling AMR by 2040 [2]. 

There are two major approaches to antimicrobial stewardship, with the most successful programs generally implementing a combination of both. The front-end or preprescription approach to stewardship uses restrictive prescriptive authority. Certain antimicrobials are considered restricted and require prior authorisation for use by all except a select group of clinicians. Clinicians without authority to prescribe the drug in question must contact the designated antimicrobial steward and obtain approval to order the antimicrobial. The front-end approach has the advantage of targeting specific antimicrobials for specific indications based on local resistance patterns and the hospital formulary antimicrobial stewardship programmes (ASPs) refer to a set of coordinated interventions designed to improve and measure the appropriate use of antimicrobials, as well as to promote the selection of both the most optimal agent and regimen in terms of dosage, duration, and route of administration [3]. There are different models for improving the quality of antimicrobial stewardship, such as the model proposed by Kimball et al., the "Three Es" of antimicrobial stewardship (TASE) and the Analysis of the Association between Stewardship Interventions and Expected Outcomes (ASIR), which in one institution achieved an average percentage reduction in admissions receiving vancomycin and piperacillin-tazobactam from 4.2% to 0.7% per month [4]. The use of ASPs, including subsequent review of prescriptions, has demonstrated an immediate reduction in the use of broad-spectrum antimicrobials [5]. In Colombia, the rational use of antimicrobials has reported post-intervention adherence of 82%, with a reduction in some broad-spectrum antibiotics and a decrease in resistant strains of P*seudomonas aeruginosa*, in contrast to an increase in extended-spectrum beta-lactamase-producing Enterobacteriaceae (ESBL) [6].

Hospitalised patients who initially receive intravenous antibiotics can safely switch to an oral equivalent on the third day of admission, once clinical stability has been established [7-9]. This conversion has many advantages, such as fewer complications, lower healthcare costs, and earlier hospital discharge. The three types of intravenous-to-oral conversion are sequential therapy, switch therapy, and reduction or step-down therapy to a lower spectrum. The appropriate use of antibiotics depends on selecting an agent capable of reaching the desired serum concentration to attack the presumed organism at the site of infection with an acceptable safety profile. Approximately one-third of all hospitalised patients who are started on intravenous antibiotics are eligible to switch to an oral equivalent [10]. A short course of intravenous treatment for 2-3 days followed by oral medication to complete the treatment is beneficial for many patients, except in cases of severe or life-threatening infections, in critically ill patients, or in the presence of contraindications for oral administration [11-16]. Oral treatment strategies have proven effective in conditions such as urinary tract infections, pneumonia, and osteomyelitis, where they are equivalent to intravenous therapies; however, the regimen chosen must be readily available in the tissues and active against the causative microorganisms. Likewise, it should have no adverse effects or have a lower probability of presenting them, so its adequate tolerance will lead to good adherence. Therefore, the objective of this study is to assess the role of changing antimicrobial therapy as a measure of implementation of the antimicrobial optimisation programme and as an audit strategy for microbiological isolates during the period from January to July 2024 in a tertiary care hospital in Colombia.

## Materials and methods

Audit-and-feedback stewardship strategy was performed, while data analysis was retrospectively collected. The study was conducted based on a review of medical records and microbiological records of patients hospitalised in a tertiary hospital in Colombia, Hospital Universitario San Rafael de Tunja, between January 1 and July 31, 2024. Variables include clinical (age, sex) and microbiological: microbiological isolation, including only one isolate per patient. Characteristics of the prescriptions were described: Antibiotic used in empiric regimen and antibiotic prescribed by ASP team, as well as the interventions on the duration of antibiotic treatment, the use of combination regimens, adherence to recommendations, and hospital readmission were included as the main outcome. Those records were collected using MS Excel (Microsoft Corporation, Redmond, Washington, United States). 

Patients over 18 years who had intervention by the ASP team were included through a prospective audit and therapeutic adjustment by the ASP team. Patients who died, were discharged, or were referred before the ASP's intervention were excluded, as well as those who did not have microbiological isolates available for review. 

The ASP program consists of a multidisciplinary team composed of an infectious disease specialist, a supporting general practitioner, a pharmacist, and a professional nurse. ASP activities include prospective auditing and feedback on antimicrobial use in the different units of the participating centre, monitoring of antimicrobial consumption, and follow-up on clinical indicators. ASP members each had a 1.0 full-time equivalent (FTE) allocation. The ASP held daily auditing rounds in which recommendations were made on the type and dose of antimicrobials that were being used in a particular patient. The treating physicians were free to follow these recommendations, but disagreements were resolved at follow-up meetings between the ASP group and the treating physician. The ASP conducted daily audit rounds during which recommendations were provided regarding the selection and dosing of antimicrobials for individual patients. Treating physicians could choose whether to implement these recommendations, and any disagreements were addressed in follow-up meetings between the ASP team and the treating physician.

Statistical analysis

Statistical analysis was performed using R version 2025.05.0+496 (R Foundation for Statistical Computing, Vienna, Austria) [17]. Quantitative variables were summarised using measures of central tendency (mean, median) and dispersion (standard deviation, minimum, maximum, and interquartile range). The normality of the distributions was assessed using the Shapiro-Wilk test.

For comparisons between groups, the Wilcoxon rank-sum test was used for nonnormally distributed quantitative variables. Categorical variables were compared using the Chi-square test with Yates's correction or Fisher’s exact test, when appropriate.

Qualitative variables were expressed as absolute and relative frequencies (percentages). Descriptive tables and graphical representations were used for presentation, including Sankey diagrams to visualize changes in antimicrobial regimens following ASP interventions, and pie charts to illustrate the proportional distribution of therapeutic adjustments.

P-values were calculated using the Wilcoxon rank-sum test for comparisons of nonnormally distributed continuous variables, and the Chi-square test with Yates's correction or Fisher’s exact test for categorical variables. Statistical significance was defined as p < 0.05.

## Results

During the observation period, 487 hospitalised patients were assessed by the ASP team. The median age was 59 years (IQR: 42-71), and 53.8% of patients were male. The main causes of hospitalisation were urinary tract infection (27.7%), skin and soft tissue infections (a broad spectrum of infections, ranging from cellulitis to necrotising fasciitis, often necessitating hospital admission and systemic antimicrobial therapy) (15.8%), and osteoarticular or musculoskeletal conditions (11.1%) (Table 1).

**Table 1:** Distribution of the main causes of hospital admission ASP: antimicrobial stewardship programme Distribution of the main reasons for hospitalization among patients treated by ASP during the study period. The most frequent causes, according to diagnosis upon hospital admission, are presented

Clinical diagnosis	Number of cases (n)	Percentage (%)
Urinary tract infection (UTI)	135	27.7
Skin and soft tissue infections	77	15.8
Osteoarticular or musculoskeletal infections	54	11.1
Gastrointestinal infections	52	10.7
Surgical site infections (SSIs) or post-surgical complications	37	7.6
Healthcare-associated infections (HAIs)	23	4.7
Upper respiratory tract infections	10	2.1
Lower respiratory tract infections	8	1.6
Central nervous system infections (CNS)	5	1
Other infectious or non-infectious diagnoses	86	17.7

Empirical use of antimicrobials

The most frequently used antimicrobials in empirical treatment were piperacillin/tazobactam (14.4%), cefazolin (12.7%), and vancomycin (10.7%). The following antibiotics were identified: ertapenem (9.5%), cefepime (8.4%), meropenem (8.4%), metronidazole (5.3%), clindamycin (3.9%), ceftriaxone (3%), trimethoprim/sulfamethoxazole (2.8%), and ampicillin/sulbactam (2.5%). It is noteworthy that 2% of patients did not receive antimicrobials in the period prior to microbiological isolation. The utilisation of piperacillin-tazobactam as a first-line empirical antimicrobial agent exhibited a decline following the implementation of the programme. Although the frequency of use at the institution remained high, even in cases of urinary tract infections, further investigation is necessary to ascertain the reasons for this phenomenon. However, the study did not include an examination of changes in the frequency of antimicrobial use.

A total of 28.7% of patients received combined antimicrobial regimens, the most common combination being vancomycin with piperacillin/tazobactam (3.5%). These combinations reflect an initial broad-spectrum empirical approach, probably aimed at covering multidrug-resistant pathogens or managing severe infections. The most frequent combinations are detailed in Table 2.

**Table 2:** Top 10 empirical antimicrobial combinations by frequency of use Frequency of use of combined empirical antimicrobial treatment regimens among hospitalized patients. The most frequent combinations are included, reflecting clinical decisions aimed at broad-spectrum coverage against serious infections or suspected multidrug-resistant pathogens

Empirical antibiotic combination	Number of patients (n)	Percentage (%)
Vancomycin + piperacillin tazobactam	17	3.5
Cefuroxime + metronidazole	13	2.7
Cefepime + vancomycin	12	2.5
Cefazolin + metronidazole	11	2.3
Ceftriaxone + vancomycin	6	1.2
Clindamycin + trimethoprim sulfamethoxazole	6	1.2
Meropenem + vancomycin	5	1
Amikacin + ertapenem	3	0.6
Ceftriaxone + metronidazole + vancomycin	3	0.6

Interventions and adherence to ASP recommendations

**Figure 1:**
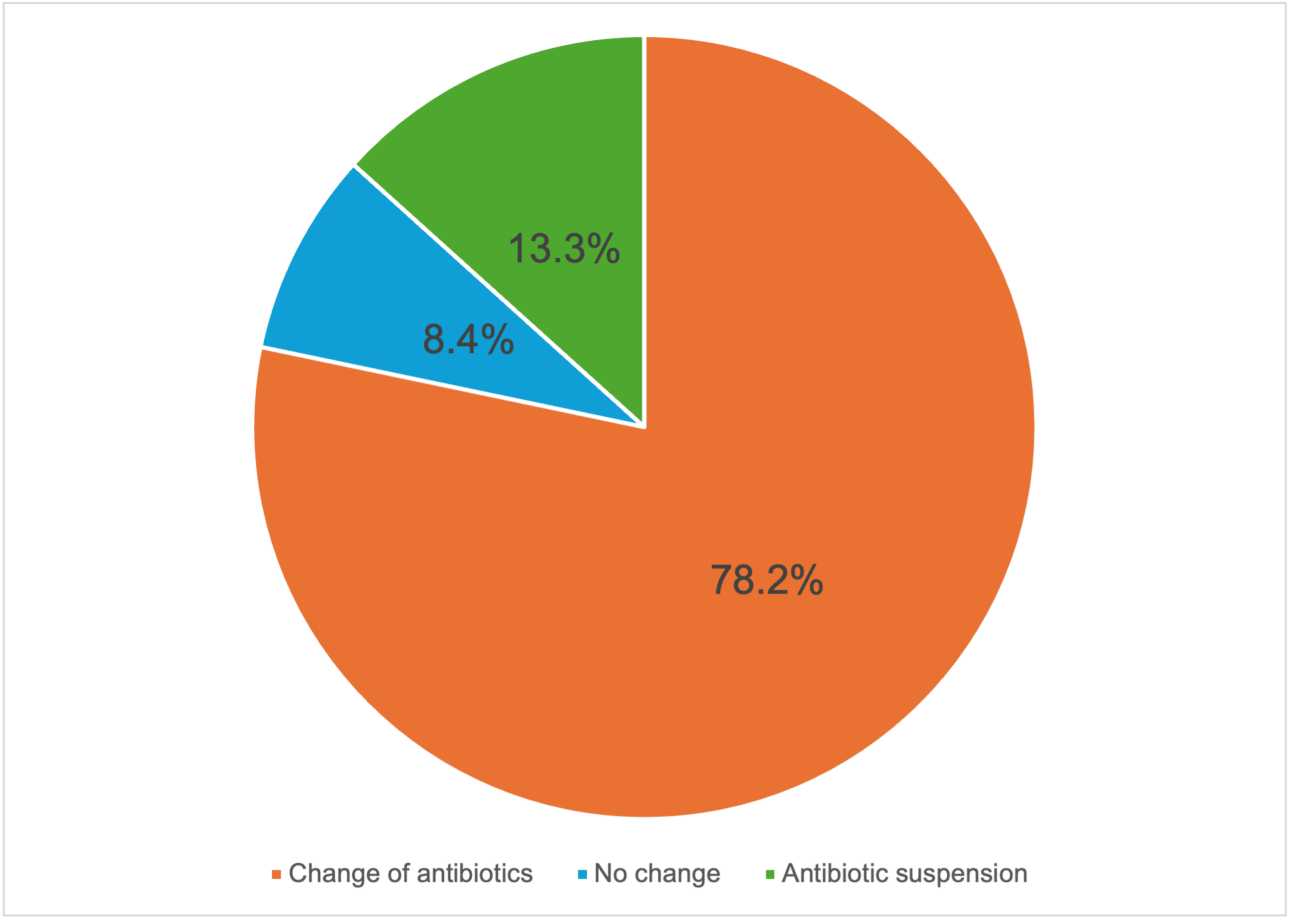
Adjustments to antimicrobial therapy following intervention by the ASPs ASP: antimicrobial optimisation programme Distribution of adjustments made to empirical antimicrobial therapy following assessment by the ASP team. This includes modifications to treatment, discontinuation, or continuation of the initial regimen, based on microbiological findings and clinical criteria

Following evaluation by the ASP team, adjustments were made to antimicrobial therapy in 78.2% of cases (n = 381), directly implemented by the ASP team. Antibiotics were discontinued in 8.4% of patients (n = 41), while in 13.3% (n = 65), no changes were made to the initial regimen (Figure 1). 

Among antibiotic change interventions, the main interventions were de-escalation (n = 176) and escalation (n = 174). Other interventions included optimising treatment duration (n = 52), initiating targeted therapy (n = 22), adjusting the dose (n = 19), discontinuing the antimicrobial (n = 14) and intervention in colonisation contexts (n = 29). In only one case, an antimicrobial was added.

Overall adherence to ASP recommendations was high (92.2%), particularly in clinical interventions such as stepwise therapy (97.7%), initiation of targeted therapy (95.5%), dose adjustment (94.7%), and optimisation of duration (94.2%). De-escalation, one of the most frequent interventions, had an adherence rate of 91.5%.

In contrast, lower levels of adherence were observed in interventions such as antimicrobial discontinuation (71.4%) and colonisation management (65.5%). These differences could reflect greater clinical complexity or resistance to implementing changes in these scenarios. Finally, the intervention corresponding to the addition of another antimicrobial agent recorded 100% adherence; however, this result corresponds to a single case and is not representative of the overall analysis.

Interventions on antimicrobial prescribing

**Figure 2:**
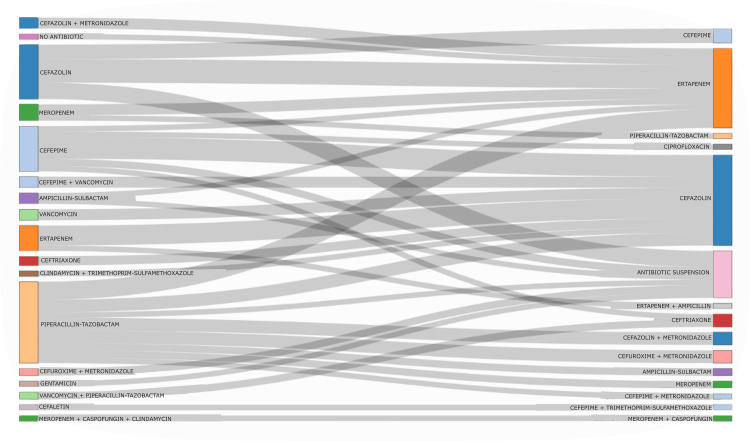
Flow of changes in empirical antimicrobial regimens following ASP intervention (Sankey diagram) ASP: antimicrobial optimisation programme Visualisation of changes in empirical antimicrobial therapy following the ASP intervention. The diagram shows the flow from initial antimicrobials to adjusted regimens, highlighting patterns of escalation, de-escalation, or change of antibiotic class

The effect of ASPs on treatment rationalisation was evident in the analysis of the adjustments made. In patients who received empirical combinations (n = 140), 74.3% (n = 104) were switched to monotherapy after the ASPs intervention. The audit allowed for a transition from broad-spectrum regimens (such as piperacillin/tazobactam) to more specific options, such as cefazolin or ertapenem, in line with microbiological results and the predominant clinical diagnosis (urinary tract infection), which optimised treatment and reduced the unnecessary use of antibiotics active against *Pseudomonas aeruginosa*. The changes in empirical and targeted therapy are shown in the Sankey diagram in Figure 2, which illustrates the therapeutic adjustment process following the prospective audit.

Microbiological isolations and resistance profiles

A total of 487 microbiological isolates were obtained, with a predominance of Gram-negative bacilli. *Escherichia coli *was the most frequent microorganism (28.3%), followed by *Klebsiella pneumoniae* (14.8%) and *Staphylococcus aureus *(14.8%). Isolates of *Pseudomonas aeruginosa* (7.2%), *Serratia marcescens *(3.9%), and *Enterococcus faecalis* (3.5%) were also identified. To a lesser extent, bacteria such as *Proteus mirabilis*, *Enterobacter cloacae* complex, *Morganella morganii*, *Staphylococcus epidermidis*, and *Citrobacter freundii *were isolated, each with frequencies between 1.4% and 3.0%. Table 3 details the microorganisms most frequently isolated in hospitalised patients included in the study.

**Table 3:** Main etiological agents identified Frequency of the main etiological agents identified in microbiological isolates obtained during hospitalisation. Gram-positive bacteria, gram-negative bacteria, and fungi are included

Microbiological isolation result	Number of patients (n)	Percentage (%)
Escherichia coli	138	28.34
Klebsiella pneumoniae	72	14.78
Staphylococcus aureus	72	14.78
Pseudomonas aeruginosa	35	7.19
Serratia marcescens	19	3.90
Enterococcus faecalis	17	3.49
Klebsiella oxytoca	15	3.08
Proteus mirabilis	14	2.87
*Enterobacter cloacae *complex	12	2.46
Morganella morganii	12	2.46

Resistance mechanisms were identified in 42.3% of isolates (n = 206). Among Enterobacteriaceae, resistance to ceftriaxone was documented in 0.5% of cases, a finding considered a phenotypic marker of ESBL production. A confirmatory test was performed using the VITEK 2 automated system, which performs BLEE identification testing. Likewise, 39 isolates (8%) were recorded as resistant to carbapenems. 

In the case of *Staphylococcus aureus*, resistance to oxacillin was observed in 24 isolates (33.3%). Furthermore, 50 isolates (10.3%) were identified as species capable of inducing chromosomal AmpC-type beta-lactamases, including *Aeromonas *spp., *Morganella morganii*, *Providencia *spp., *Citrobacter *spp., *Hafnia *spp., *Enterobacter cloacae*, *Klebsiella aerogenes*, and *Serratia *spp.

Hospital stay

Hospital stays were analysed according to the type of intervention performed by the ASP, the results of which are presented in Table 4. Patients with de-escalation had a median of 11 days (mean: 17.1; SD: 20.0), while those with escalation had a median of 19.5 days (mean: 27.7; SD: 25.0). The initiation of targeted therapy and optimisation of treatment duration showed the highest mean hospital stays (42.9 and 33.1 days, respectively), which could reflect the underlying clinical complexity.

**Table 4:** Length of hospital stay according to intervention by the antimicrobial stewardship programme *NC: not calculated; PROA: programs for optimizing the use of antibiotics Comparison of hospital stay days between patients according to the PROA intervention. Measures of central tendency and dispersion are presented

Type of intervention	Days of stay					
	# cases	Average	Median	DE	Min	Máx
Therapeutic de-escalation	176	17.1	11	20	0	123
Therapeutic escalation	174	27.7	19,5	25	2	123
Optimisation of treatment duration	52	33.1	16,5	36.2	3	137
Intervention due to colonisation	29	19.2	13	15.2	2	56
Commencement of targeted therapy	22	42.9	41	30.9	3	123
Dose adjustment	19	14.4	15	11.7	3	54
Suspension of antimicrobials	14	21.6	20,5	17.9	3	72
Addition of another antimicrobial agent	1	42	42	NC	42	42

Based on the adherence to the recommendations issued by ASPs, in the adherence group (n = 449), the median length of stay was 16 days, with a mean of 24.8 days (SD: 25.5; range: 0-137), and in patients whose prescribers did not follow the recommendations (n = 38), the median length of stay was nine days, with a mean of 14.4 days (SD: 14.4, RIC 3-63). It should be noted that the adherence group accounts for most of the sample, which could influence the distribution and average length of hospital stay. Therefore, any comparison between these groups should be interpreted with caution.

Duration of antibiotic treatment

The days of empirical antibiotic treatment (before microbiological isolation) and targeted treatment (after ASP intervention) were evaluated. The data did not follow a normal distribution (Shapiro-Wilk test, p < 0.001), so the Wilcoxon test was used. No significant differences were found in the duration of empirical treatment between the groups with and without intervention (median: 2 days; p = 0.36). However, targeted treatment was significantly shorter in patients who underwent intervention by the ASP team actively (median: 5 days) compared to those who did not (median: 7 days; p < 0.001), suggesting a favourable impact of ASP on optimising antimicrobial use.

Hospital readmissions

At the 30-day follow-up after hospital discharge, an overall readmission rate of 19.1% (n = 93) was recorded. The proportion was slightly higher among patients who underwent intervention and therapy adjustment (19.4%) compared to those without changes in their regimen (17.8%). However, these differences were not statistically significant (Yates-corrected chi-square: p = 0.838; Fisher's exact test: p = 0.882), and the comparison is limited by the smaller number of cases in the group without adjustment (n = 90).

## Discussion

The fundamental purpose of ASPs is to optimise the use of antimicrobials to improve clinical outcomes for patients [3], while minimising the adverse effects associated with their use, such as toxicity, the selection of resistant bacteria or the emergence of *Clostridioides difficile *infections. In this context, graphical analysis of therapeutic transitions is a key tool for visualising the effects of program interventions, such as antibiotic de-escalation, escalation, rotation, or discontinuation [18-20]. In the present study, the 20 most frequent combinations of changes in antimicrobial therapy were represented using this visualisation, which clearly showed the transitions between the initial empirical regimens and the therapies adjusted after the audit, facilitating the identification of common prescribing patterns and opportunities for improvement, such as the discontinuation of combinations that have utility or impact on AMR and adverse effects, even in critically ill patients [21-23].

Among the main types of interventions carried out during the study, four stand out. First, therapeutic de-escalation refers to a safe and effective strategy for treating various types of infections, consisting of reducing the spectrum of empirical antimicrobial treatment according to microbiological findings [21]. The current study identified shifts from broad-spectrum antibiotics such as meropenem, ertapenem, cefepime, and piperacillin/tazobactam to narrower-spectrum antimicrobials such as cefazolin or even to antibiotic discontinuation. These transitions are considered a key indicator of ASP success [24], as they involve the use of the most specific and less aggressive antibiotic possible once the pathogen and its sensitivity have been identified or if clinical improvement is observed [23]. 

Secondly, treatment escalation, where there was a shift from narrow-spectrum antibiotics to broader-spectrum antimicrobials, such as from cefazolin to ertapenem or cefepime. Although the main objective of ASP is to promote de-escalation, this intervention may be necessary in the event of therapeutic failure, clinical deterioration of the patient, or isolation of a resistant pathogen, which may be an effect of the ASP intervention without necessarily implying malpractice or intervention, as it reflects the reality of the clinical status of patients at the time of the study.

Thirdly, rotation or change for optimisation: adjustments were identified between antibiotics with a similar spectrum or between therapeutic combinations, motivated by multiple clinical or microbiological factors. The most frequent reasons included culture and antibiogram results that justified a change in treatment, as well as the appearance of adverse effects to the initial antibiotic, cost-effectiveness considerations, the presence of drug interactions, and strategies aimed at reducing the selection pressure that favours bacterial resistance [25]. These types of adjustments reflect ASP's active involvement in the individualisation and continuous improvement of antimicrobial treatment. 

Finally, the suspension of antibiotic treatment and transitions from antibiotic use to antibiotic suspension represent a very positive outcome for ASP. They indicate that treatment was interrupted in a timely manner, either due to clinical resolution, diagnosis of nonbacterial aetiology, or completion of the therapeutic cycle. Adequate treatment duration is one of the cornerstones of ASP, as it contributes significantly to reducing unnecessary exposure to antimicrobials, with extensive studies showing that short treatments are just as effective in common infections [26-28]. 

The low frequency of resistant microorganisms with low detection of ESBL and carbapenem resistance in Enterobacteriaceae, as well as oxacillin-resistant *S. aureus* strains, suggests a favourable microbiological profile that does not warrant the use of broad-spectrum therapies in most empirical treatments. Similarly, this has been evaluated even in immunocompromised patients [29], where there are no differences in mortality; however, the use of narrower spectrum therapies can be linked to higher readmission rates, as evidenced in the present study. Similarly, the presence of species with AmpC induction potential reinforces the importance of appropriate antibiotic selection, since inappropriate use of cephalosporins may favour the expression of these mechanisms. Taken together, these findings justify early ASP intervention, supporting its role in containing resistance and reducing the unnecessary use of broad-spectrum antimicrobials. It also reinforces the concept that clinical status does not necessarily determine the finding of resistant strains [30]. Furthermore, it does not justify the prescription of broad-spectrum antibiotics in all clinical scenarios.

Finally, in order to establish a proactive strategy for the future, the audit adherence was not classified in each of the services offered in the health institution, such as emergency care, hospitalisation, intensive care unit, among others; nor was adherence classified by speciality, which would have allowed a comparison between areas and their exposure to types of antimicrobials, type of dosage and duration of therapy, which could be studied in a qualitative approach [31]. Likewise, despite the implementation of ASP strains, AMR continue to be identified, and, therefore, studies are needed to assess the impact of inappropriate prescribing in outpatient settings or in referral centres of more complex hospitals [32].

In the study by Duane et al., which evaluated more than 2,300 interventions from a stewardship program at a high-complexity university hospital, the overall rate of compliance with recommendations was significantly lower in surgical services (70%) than in medical services (83.5%), a difference that remained consistent for both “appropriate coverage” interventions (82.1% vs. 90.5%) and those aimed at reducing selective pressure (69.5% vs. 81.1%). This finding suggests a consistent tendency for surgical practitioners to more readily accept recommendations involving adding or maintaining antibiotic coverage, but to show greater resistance to therapeutic reduction or de-escalation. Factors proposed to explain this lower surgical adherence include the persistence of prolonged empirical regimens due to fear of postoperative infectious complications, a heightened perception of prescriptive autonomy, and resistance to unsolicited recommendations [33]. Finally, there were barriers to our study in terms of the limited availability of studies in low-income countries that demonstrate the impact of ASP implementation on health institutions, the health system, and the environment [34]. It should be noted that, as it was a retrospective study, selection bias (only ASP-reviewed patients included), lack of adjustment for infection severity, absence of mortality analysis and a single-centre study design are among its limitations. It was not possible to determine the clinical impact and progression of the disease in terms of treatment success in the patients evaluated. Probably because there were infections associated with healthcare, and although our objective was to evaluate the role of changing antimicrobial therapy as a measure of implementing the antimicrobial optimisation programme, it was not possible to verify the impact of ASP on the resolution of collateral infections in a hospitalisation context.

## Conclusions

This study reflects the reality and complexity of implementing ASP in a tertiary care institution in Colombia. However, it shows the benefits of implementing this type of programme and how it promotes the use of narrower-spectrum antimicrobials with a greater transition from anti-*Pseudomonas aeruginosa *antimicrobials to first-generation cephalosporins. Although there were no differences in readmissions or significant clinical variables such as mortality, these were not included since the adjustment of antimicrobials is probably not the only factor impacting these variables, and biases may arise in their interpretation.
